# Bacteriophage Challenge Drives Genomic and Proteomic Remodeling and Alters Antibiotic Resistance in a Methicillin-Resistant *Staphylococcus aureus*

**DOI:** 10.3390/antibiotics15080800

**Published:** 2026-08-18

**Authors:** Otília Vágó, Krisztián Laczi, László Orosz, Georgina Horváth, Károly Péter Sárvári, Diána Szabó, Regina Csordás, Zoltán Szabó, Caleb Ardizzone, Titanilla Szögi, Katalin Burián, Dezső Péter Virok

**Affiliations:** 1 Pándy Kálmán County Hospital, Semmelweis Str. 1, H-5700 Gyula, Hungary; 2 Department of Biotechnology and Microbiology, University of Szeged, Közép fasor 52, H-6726 Szeged, Hungary; 3 Department of Medical Microbiology, Albert Szent-Györgyi Health Center and Albert Szent-Györgyi Medical School, University of Szeged, Semmelweis Str. 6, H-6725 Szeged, Hungaryhorvath.georgina@med.u-szeged.hu (G.H.); burian.katalin@med.u-szeged.hu (K.B.); 4 Department of Oto-Rhino-Laryngology and Head & Neck Surgery, University of Szeged, Tisza Lajos Str. 111, H-6724 Szeged, Hungary; 5 Department of Medical Chemistry, Albert Szent-Györgyi Medical School, University of Szeged, Dóm Sq. 8, H-6720 Szeged, Hungary; 6 Division of Infectious Diseases, Department of Medicine, Indiana University School of Medicine, 635 Barnhill Drive, MS 258, Indianapolis, IN 46202, USA; calardiz@iu.edu; 7 Institute of Pathology, University of Szeged, Állomás Str. 1, H-6725 Szeged, Hungary; szilagyi-szogi.titanilla.zita@med.u-szeged.hu

**Keywords:** bacteriophage, cocktail, antibiotic, resistance, *Staphylococcus*, MRSA, genomics, proteomics, PYOFAG

## Abstract

**Background/Objectives**: Methicillin-resistant *Staphylococcus aureus* (MRSA) is a major therapeutic challenge due to its extensive antibiotic resistance. This study examined how bacteriophage exposure affects the genome, proteome and antibiotic susceptibility of a clinical MRSA isolate. **Methods**: The MRSA isolate was exposed to the PYOFAG bacteriophage cocktail, and small colony variants (SCVs) of the surviving bacteria were compared with the untreated parental strain by phenotypic testing, whole-genome sequencing, comparative proteomics, and transmission electron microscopy. **Results**: Phage exposure induced marked remodeling in MRSA, including altered growth, colony morphology, and increased susceptibility to various antibiotics, especially β-lactams and aminoglycosides. Genomic analysis identified multiple mutations and the loss of two genomic regions, including changes in *tarS*, a gene linked to wall teichoic acid glycosylation, phage adsorption, and β-lactam resistance. Proteomic analysis revealed broad changes in metabolic, stress-response, and cell-envelope-associated pathways. Transmission electron microscopy showed a significant reduction in cell wall thickness after phage treatment. **Conclusions**: Bacteriophage exposure drives phenotypic and molecular adaptation in MRSA and may create evolutionary trade-offs that weaken resistance mechanisms. These findings support the potential of bacteriophages as both direct antibacterial agents and modulators of antibiotic susceptibility.

## 1. Introduction

MRSA represents a significant clinical challenge due to its role as a leading cause of nosocomial infections (hospital-associated MRSA or HA-MRSA), and is also a prominent public health concern in community contexts (community-associated MRSA or CA-MRSA) [1]. Treatment is challenging due to its resistance to conventional antibiotics. The primary mechanism underlying MRSA resistance to methicillin and other β-lactam antibiotics is due to the *mecA* gene, which encodes PBP2a, a modified penicillin-binding protein with low affinity for most β-lactam antibiotics. MRSA has been observed to develop resistance to non-β-lactam agents such as macrolides, clindamycin, and fluoroquinolones [2,3].

Bacteriophages are viruses that selectively infect and lyse bacterial cells through specific receptor-mediated interactions. Compared with antibiotics, their advantages include selective targeting of pathogenic bacteria, the ability to kill antibiotic-resistant strains, and the capacity to penetrate and disrupt bacterial biofilms that typically protect microbes from conventional antimicrobial agents. Therefore, bacteriophage therapy is gaining traction as an alternative to conventional antibiotics. Researchers have characterized specific bacteriophages that exhibit lytic activity against MRSA, disrupt biofilms, and reduce infection severity [4,5]. Bacteriophage therapy, in conjunction with antibiotics, has also shown promise against MRSA in certain clinical settings [6].

Phage-antibiotic synergy (PAS) refers to the enhanced killing of bacteria when a phage and an antibiotic are used in combination. The PAS effect has been shown against various bacteria, such as *Escherichia coli* [7], *Klebsiella pneumoniae* [8], *Pseudomonas aeruginosa*, and *Acinetobacter baumannii* [9]. The treatment of MRSA with the combination of phages and antibiotics like daptomycin and ceftaroline has shown synergistic effects, leading to reductions in bacterial loads in both in vitro and ex vivo models [10,11]. Phage-antibiotic combinations have also been effective in disrupting MRSA biofilms that pose a significant treatment challenge in clinical settings [12]. An interesting additional impact of bacteriophages could be the reversal of antibiotic resistance. If bacteria survive phage infection, an evolutionary trade-off can occur in which the adaptations that confer phage resistance come at the cost of reduced antibiotic resistance. We previously showed that phage infection/exposure altered the proteome and colony morphology of an ESBL *Escherichia coli* isolate and increased its sensitivity to various antibiotics, including β-lactams [13]. Tran et al. showed that infection by the lytic phage ΦStaph1N and its mutant derivative Evo2 caused MRSA strains to become sensitized to β-lactam antibiotics such as oxacillin and cefazolin [14].

In this study, we investigated the impact of a commercially available bacteriophage cocktail on the colony morphology, genome, proteome, and antimicrobial susceptibility of an MRSA isolate. Consistent with our previous findings [13], phage exposure induced substantial metabolic reprogramming in MRSA, which was associated with alterations in molecular and phenotypic traits including altered antibiotic resistance.

## 2. Results

### 2.1. Bacteriophage Infection Changes the Colony Morphology and Growth of the Clinical Isolate MRSA_US14_

**Figure 1:**
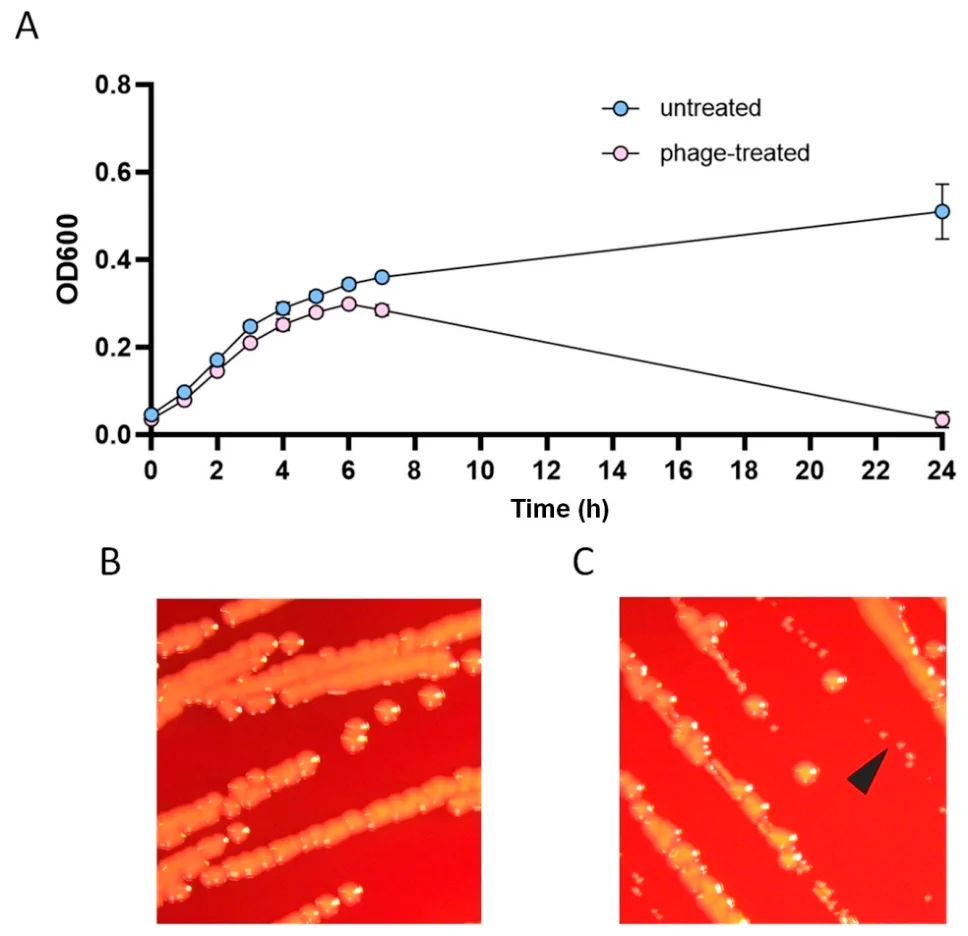
Impact of bacteriophage exposure on the growth and colony morphology of MRSA_US14_. (**A**) Growth kinetics of untreated and phage-treated MRSA_US14_ in LB broth. Colony morphology of (**B**) untreated and (**C**) phage-treated MRSA. The arrow shows the SCV MRSA visible after 24 h of phage treatment.

Approximately 3.2 × 10^6^ *S. aureus* cells (80 µL) were incubated with a PYOFAG phage cocktail (20 µL) or left untreated for 1, 2, 3, 4, 5, 6, 7, and 24 h at 37 °C and 5% CO_2_. The exact multiplicity of infection (MOI) could not be precisely determined due to the proprietary composition of the cocktail; however, the manufacturer reports a concentration exceeding 10^5^ phage particles per mL, comprising phages targeting multiple bacterial species and strains. The untreated MRSA_US14_ culture shows a typical growth profile over 24 h, with a rapid increase in optical density during the first 7 h followed by continued biomass accumulation through the 24 h time point (Figure 1A). In contrast, the phage-treated culture initially tracks closely with the untreated control, displaying a comparable early rise in OD_600_ till 6 h post infection. This early overlap indicates that phage exposure does not immediately suppress bulk culture turbidity, which is consistent with a delay between adsorption/infection and population-level lysis. After 6 h, the trajectories diverge sharply: the phage-treated culture exhibits a progressive decline in OD_600_ across the remainder of the experiment, reaching near-baseline values by 24 h. The sustained decrease in turbidity is most consistent with extensive phage-mediated killing and lysis dominating over bacterial replication. After 24 h of incubation, the cultures were plated on Columbia blood agar, and colony morphology from overnight cultures (37 °C, 5% CO_2_) was assessed. The untreated MRSA_US14_ produced characteristic large, yellowish colonies (Figure 1B). Interestingly, viable phage-treated MRSA were still recovered upon plating on blood agar. Notably, many colonies were substantially smaller than those from the untreated control, indicating enrichment of slow-growing survivor subpopulations (Figure 1C). This phenotype is consistent with SCV-like outgrowth and/or fitness costs associated with phage resistance following phage-mediated stress.

### 2.2. Bacteriophage Exposure Alters the Antimicrobial Resistance of MRSA_US14_

**Figure 2:**
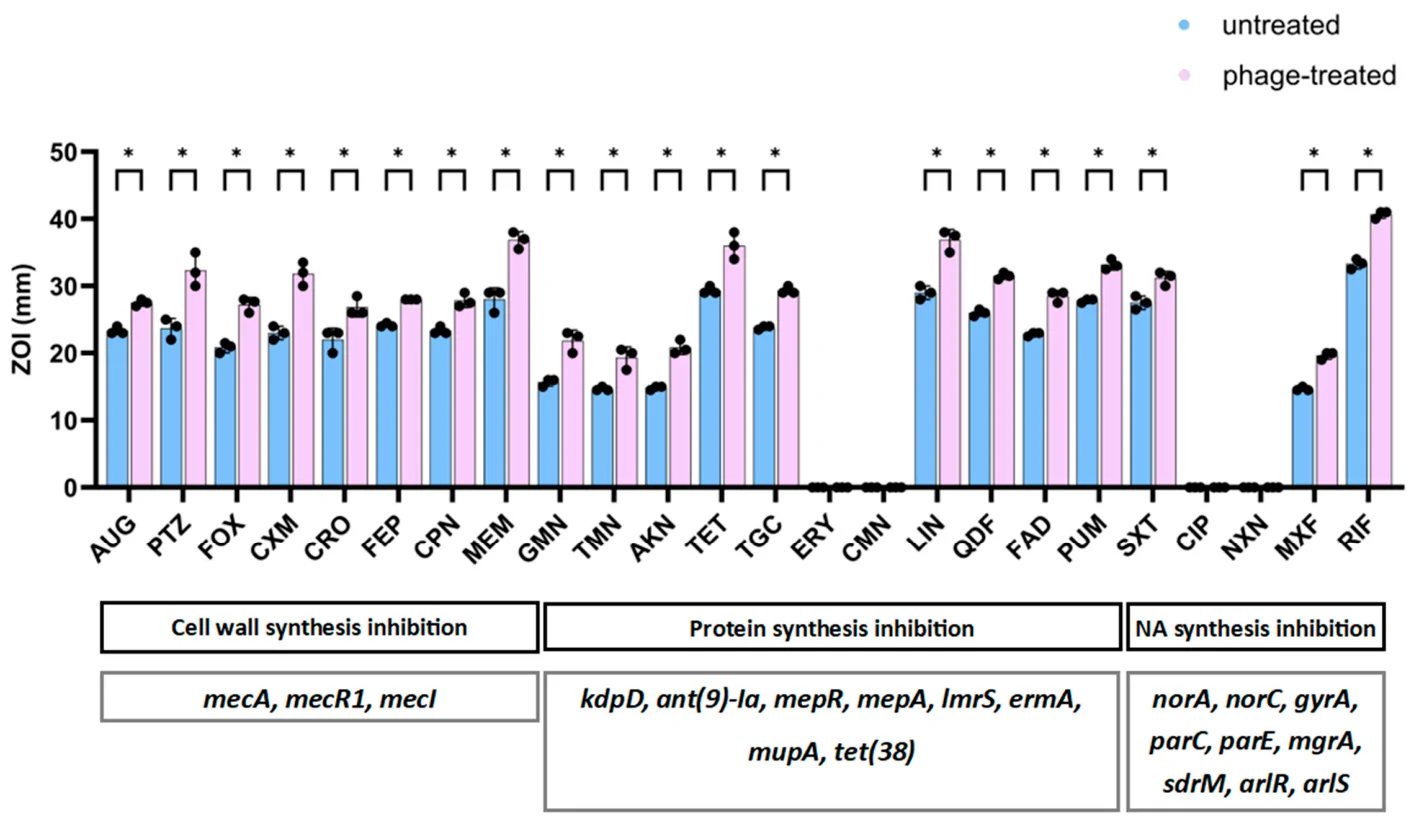
Impact of bacteriophage exposure on the antibiotic resistance of MRSA_US14_. Disk diffusion test-based antibiotic resistance measurements of untreated (blue) and phage-treated (pink) *S. aureus*. Diameters of zones of inhibition (ZOI) are shown (*n* = 3). Adjusted *p* values were calculated by the Holm-Šídák method. * *p* < 0.05. The antibiotic mechanisms of action and the antibiotic resistance genes detected by Proksee [16] in the MRSA_US14_ genome are also shown. Abbreviations: NA, nucleic acid.

We compared the antibiotic susceptibility of untreated MRSA_US14_ and the phage-treated SCV MRSA_US14_. Bacteriophage exposure resulted in a consistent and often substantial increase in antibiotic susceptibility across most tested agents, as reflected by the percentage change in inhibition zone diameters (Figure 2). Interestingly, we observed an apparent β-lactam susceptibility of the untreated MRSA_US14_ isolate in the disk diffusion assay. These results should be interpreted with caution, as testing was performed on Columbia blood agar rather than standard Mueller–Hinton (MH) agar due to the poor growth of the phage-treated MRSA_US14_ on MH agar. Previous studies have shown that blood-containing agar can impair the detection of methicillin/oxacillin resistance in *Staphylococcus aureus* compared with MH agar, potentially leading to underestimation of resistance [15].

The most pronounced increases in susceptibility were observed among β-lactam antibiotics and aminoglycosides. Within the β-lactam class, cephalosporins such as cefuroxime (CXM, +38.41%), cefoxitin (FOX, +30.72%), and ceftriaxone (CRO, +21.97%) showed marked increases in susceptibility, while the β-lactam/β-lactamase inhibitor combinations piperacillin–tazobactam (PTZ, +36.62%) and amoxicillin–clavulanate (AUG, +17.86%) also exhibited notable improvements. Aminoglycosides displayed some of the largest relative increases overall, with amikacin (AKN, +40.45%), gentamicin (GMN, +39.36%), and tobramycin (TMN, +31.82%) all showing strong phage-associated sensitization. Several additional antibiotic classes also demonstrated moderate susceptibility gains, including tetracycline (TET, +22.73%), tigecycline (TGC, +23.08%), fusidic acid (FAD, +24.82%), linezolid (LIN, +27.01%), and rifampicin (RIF, +22.12%). These broad-spectrum increases suggest that phage exposure may induce global physiological changes rather than affecting a single resistance pathway. In contrast, antibiotics to which the isolate was originally completely resistant, specifically ERY, CMN, CIP, and NXN, showed no measurable change, suggesting that phage treatment does not universally overcome antibiotic resistance.

### 2.3. Bacteriophage Exposure Alters the Genome of MRSA_US14_

To gain deeper insight into genomic changes, we sequenced the genomes of untreated MRSA_US14_ and phage-treated SCV MRSA_US14_. A total of 2.6 and 2.8 million read pairs were generated in the sequencing reaction from the untreated and the phage-treated samples, respectively. Ninety-six per cent of the reads survived quality trimming and were used in the assembly. The assembly resulted in 45 contigs in the untreated and 46 contigs in the phage-treated sample, with a total length of 2,807,391 base pairs (bp) and 2,808,531 bp, respectively. Both assemblies had an N50 greater than 150,000 bp, meaning that at least half of each assembled genome was contained in contigs of 150 kb or longer, and an L50 of 5, indicating that only the five largest contigs accounted for 50% of the total assembly length. Additionally, 99.9% of the reads could be mapped back to them. The GC content was 32.71%. Based on CheckM2 results, the completeness of both genomes was 100% with less than 1% contamination. The two genomes shared 99.29% identity, and 2,788,943 bases could be aligned between them.

**Figure 3:**
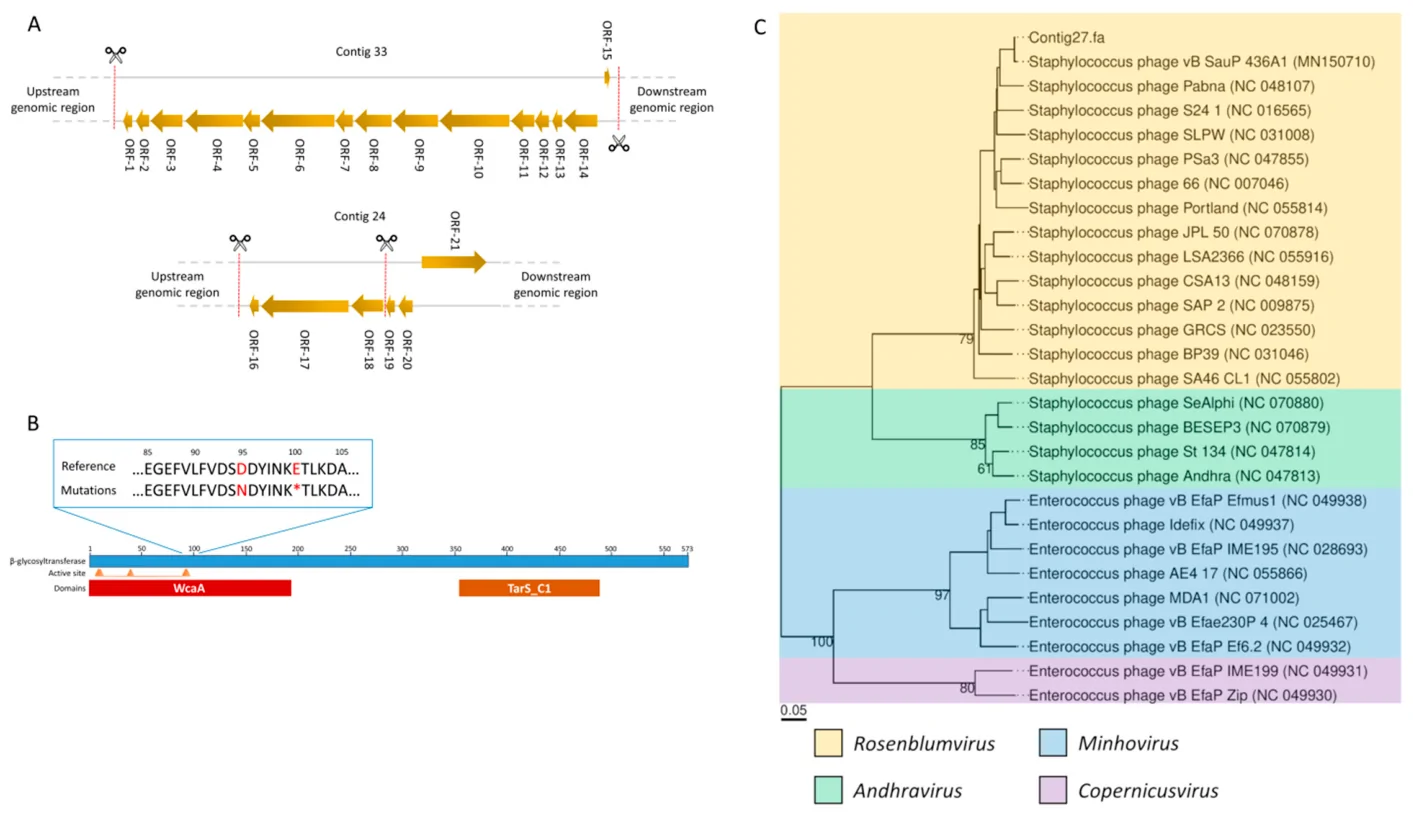
Genomic rearrangement of MRSA_US14_ under phage selection pressure and identification of the infecting phage. (**A**) Genomic regions lost as a result of phage exposure. The red dotted lines surround the lost regions. (**B**) The location of high-impact mutations in protein sequences. (**C**) The GBDP tree of *Rountreeviridae* genomes generated by VICTOR. The tree was inferred using formula D6. The numbers above the branches are GBDP pseudo-bootstrap support values from 100 replications. The branch lengths of the resulting VICTOR trees are scaled in terms of the respective distance formula used. The color codes represent different viral genera belonging to this family based on the NCBI Taxonomy database.

We detected multiple differences between the phage-treated and untreated genomes. Two contigs (24 and 33) present in the untreated control are completely or partially missing from the phage-treated strain. The missing regions showed no coverage in the phage-treated strain. Contig 33 is a 14,114 bp long contig harboring 15 genes mainly coding for proteins associated with the type IV secretory system (T4SS) of DNA conjugation (Figure 3A). The following proteins encoded on the contig could be identified with blastp, eggnog-mapper, CDD and/or SMART: ORF1: hypothetical protein resembling a partial TraO, ORF2: single-stranded DNA-binding protein (Ssb), ORF3: TrbL family conjugal transfer protein, ORF4: T4SS-associated conjugal transfer protein (VirD4-like), ORF5: hypothetical protein (putative TraJ), ORF6: DNA topoisomerase III (TopB/TraE), ORF7: periplasmic protein associated with T4SS TrsH/TraH, ORF8: Mannosyl-glycoprotein endo-beta-N-acetylglucosaminidase, ORF9: WXG100 protein secretion system (Wss) protein (YukC superfamily), ORF10: VirB4 family T4SS protein, ORF11: conjugative transfer protein (TrsD/TraD), ORF12: Cag pathogenicity island type IV secretory system (CagC), ORF13: hypothetical protein, ORF14: conjugative transfer protein (TrsA), and ORF15: regulator protein. The orientation of ORF15 is antiparallel to the other genes. The arrangement and function of the genes strongly suggest they are in an operon. There is no evidence on contig 33 whether it is part of a conjugative plasmid or is built into the chromosome as a transposable element. A 4124 bp long chunk of contig 24 did not have an equivalent in the phage-resistant strain (Figure 3A). This region covers three genes: ORF16: fructoseamine kinase (Pck-like superfamily), ORF17: Isoleucine-tRNA (IleS) synthase, and ORF18: transposase (IS6 family). ORF18 is partially deleted, and a 220 bp long chunk remained on the contig at its 5′ end. Notably, the coverage of the two lost regions dropped to 15% of the assembly’s average coverage in the parental genome. This suggests that the untreated strain is also heterogeneous with respect to these regions.

To further characterize genomic changes at the sequence level, we performed variant analysis. Variant analysis revealed 197 mutations, comprising 156 single nucleotide polymorphisms (SNPs), 37 multiple nucleotide polymorphisms (MNPs) and 4 insertions/deletions (INDELs). Fifty-five mutations were intergenic, and 28 genes were affected by one or multiple mutations. The frequency of the mutations ranged between 25.2 and 77.2%, with a mean value of 45.6%. From the mutations affecting protein-coding sequences, 85 were synonymous, 51 were missense, and four codons were converted to stop codons. One in-frame insertion was also detected. The highest-impact mutations are those that introduce stop codons, abruptly terminating the protein sequence. The cell-wall teichoic acid-modifying enzyme Poly(ribitol-phosphate) beta-N-acetylglucosaminyltransferase (TarS) acquired a stop codon (Glu100*) in the middle of the catalytic domain with a frequency of 28.8% (Figure 3B). We observed two other mutations, Asp95Asn and Ser153Arg, at 37.2% and 30.2% frequencies, respectively. Asp95Asn disrupts the catalytic site, neutralizing one of the two conserved aspartic acids in the region, while Ser153Arg may affect the domain structure by introducing a large positively charged amino acid instead of the small, slightly negative serine. These mutations are predicted to lower or disrupt the catalytic activity of the protein; therefore, they may be maintained by different cells in the population. This interpretation is also supported by the coverage data. The locus of the two mutations responsible for Asp95Asn and Glu100* is covered by the same subpopulation of the reads, but the two mutations never appear on the same read. Another stop codon was introduced in the *prmC* gene coding for the Release factor glutamine methyltransferase (PrmC). The mutation (Arg24*) occurred with 42.6% frequency near the N-terminus of the protein, eliminating it. We identified a moderate-impact mutation in the target protein of PrmC peptide chain release factor 1 (Pfr1). The Ile304Asn mutation occurred at 47.0% frequency and changed the hydrophobic isoalkyl side chain to a polar amid group. This change can influence the structure and catalytic activity of the Prf1 protein. Additional stop codons were identified in a repA gene of a 15kb plasmid-derived contig near the end and in a gene coding for a small 60 amino acid-long hypothetical protein with unknown function. The Gln144* mutation in RepA was at 37.9% frequency and did not affect the plasmid’s coverage in the phage-treated genome. Therefore, it is unlikely to have a significant effect. Despite its short length, trypsin-like serine protease (SplF) sustained 7 mutations (Ser17Thr, IleIle21ValVal, His36Arg, Ile42Met, GlyAsn45SerAsp, Ser49Thr, Gly55Ser) with frequencies between 35.5 and 46.4%. The surface protein SdrH suffered multiple mutations (Pro141Leu, LysProAsp127AsnProAsn, Asn123Lys). We observed several missense mutations in other surface proteins involved in adhesion. Although only one may have a major effect on the structure or function of surface protein G (SasG), the mutation Glu564Lys has a 31.1% frequency and changes the charge from negative to positive in an interdomain region, which may affect protein conformation. Another, less drastic mutation also occurred in the same region, Thr503Ala, with a higher frequency of 49.7%.

An 18,136 bp long contig harboring 19 genes is unique in the phage-treated assembly. This contig shares a 99.96% identity with the MN150710 genome based on a BLASTn search in the NCBI nt database. 14,323 reads support the contig, and the average coverage depth is 106×, which is only 39.5% of the assembly’s average coverage (268×). The 39.5% average coverage represents approximately 0.395 phage genome equivalents per bacterial genome equivalent. Assuming approximately 30 phage genome copies per actively infected cell [17], this ratio would be consistent with an actively infected subpopulation of approximately 1.3% of the bacterial cells. The phage genome belongs to the *Rosenblumvirus* SCH1 isolate *Staphylococcus* phage vB_SauP-436A1. This phage is a member of the *Rountreeviridae* family of *Caudoviricetes*. The GBDP tree (Figure 3C) supports BLAST results clustering MN15710 together with contig 27.

### 2.4. Bacteriophage Infection Alters the Proteome of MRSA_US14_

**Figure 4:**
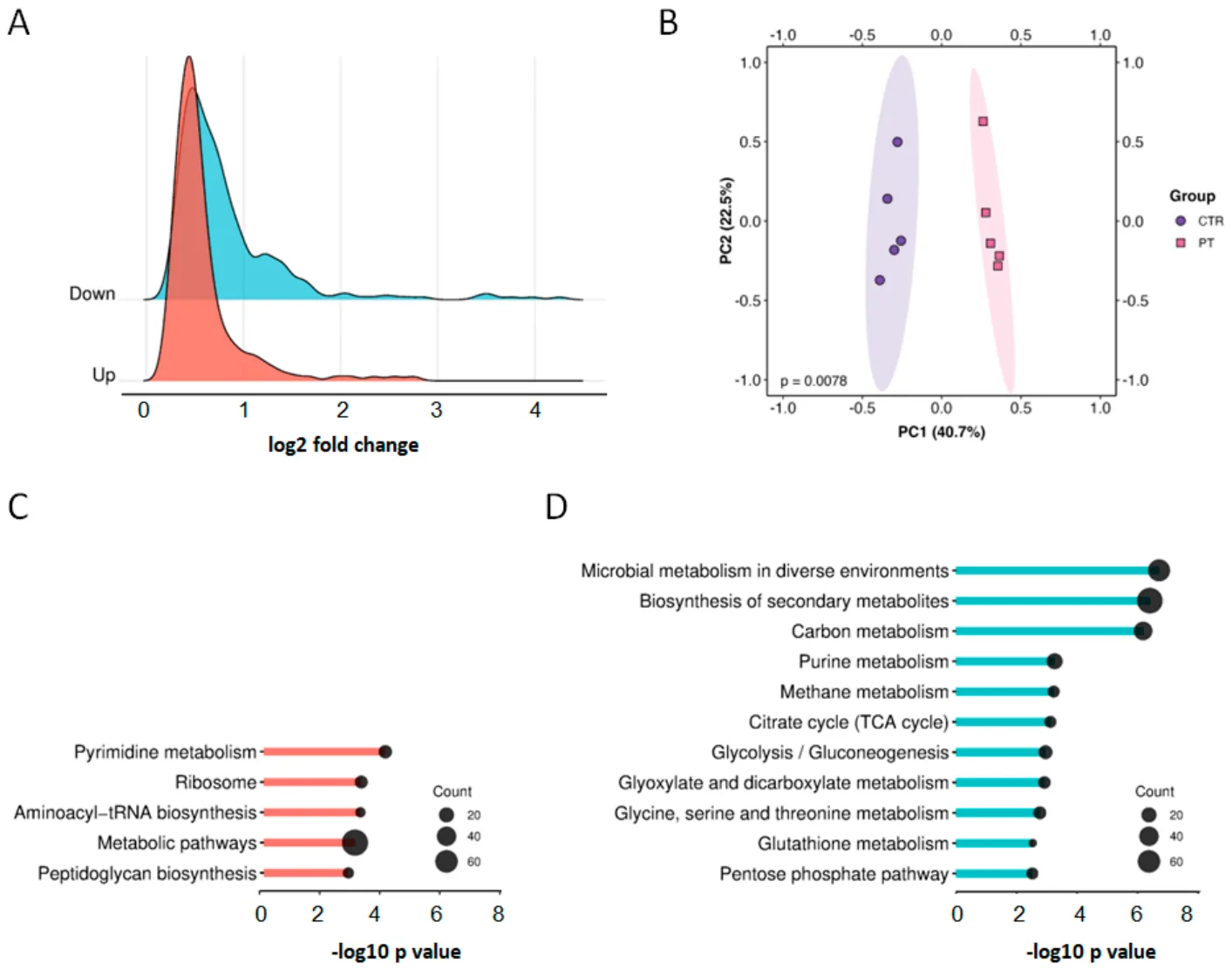
Global changes in the proteome of bacteriophage-treated MRSA_US14_. (**A**) Density plot showing the fold change distribution of upregulated (log2 fold change) and downregulated (−log2 fold change) proteins in the bacteriophage-treated MRSA_US14_. (**B**) Principal component analysis of the protein abundance data from the five biological replicates of bacteriophage-treated and untreated MRSA_US14_ samples (*n* = 5). Functional analysis of the MRSA_US14_ proteins induced (**C**) or repressed (**D**) by the bacteriophage infection. Significantly enriched (FDR Benjamini–Hochberg q < 0.05) Kyoto Encyclopedia of Genes and Genomes (KEGG) pathways among the up- and downregulated proteins identified by ShinyGo [18].

**Figure 5:**
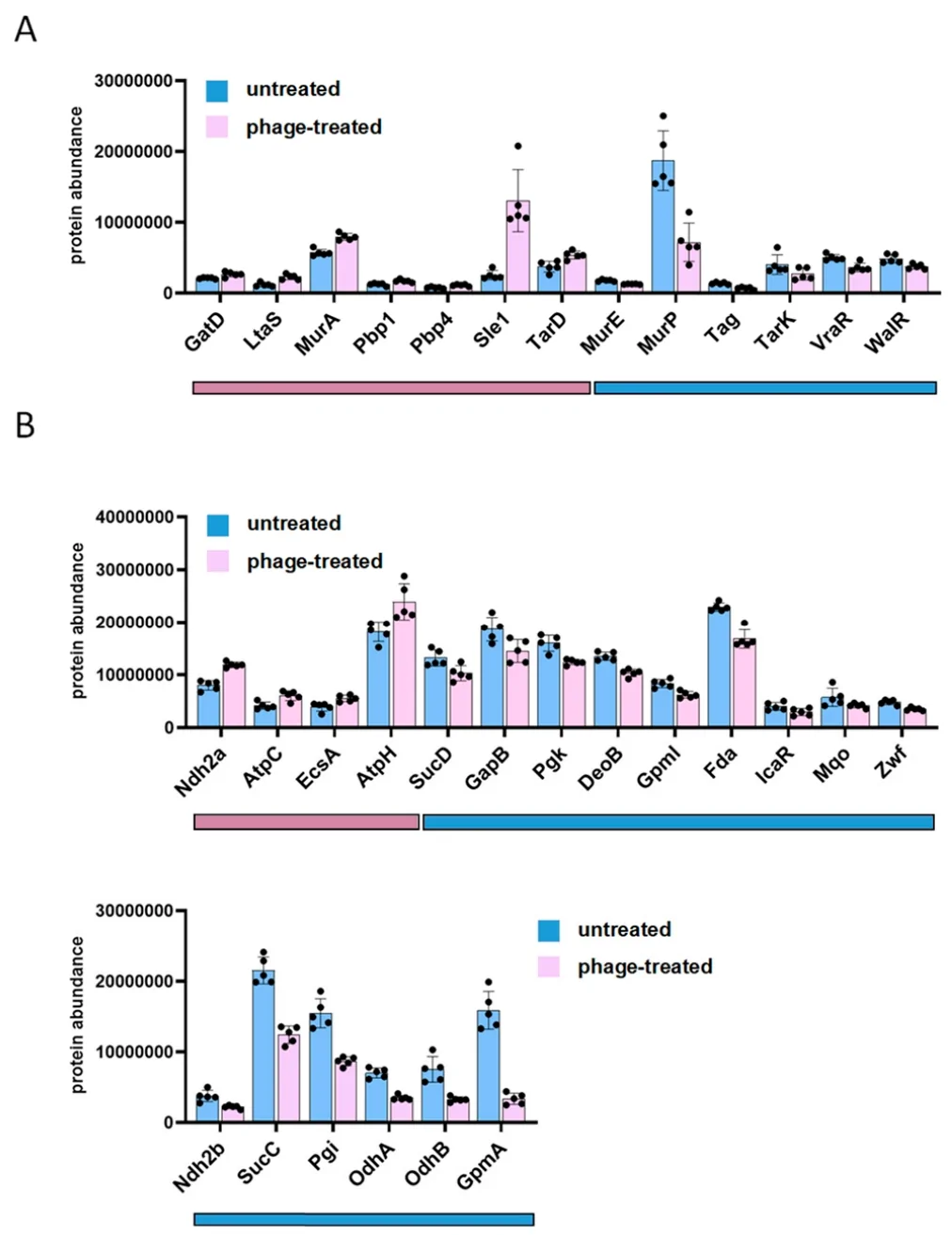
Differential regulation of MRSA_US14_ proteins associated with β-lactam and aminoglycoside resistance following bacteriophage exposure. Literature-based list of significantly downregulated and upregulated proteins potentially involved in (**A**) β-lactam and (**B**) aminoglycoside antibiotic resistance. Upregulated and downregulated proteins are indicated by red and blue horizontal bars below the x-axis, respectively.

To obtain a broader view of bacteriophage-induced phenotypic changes, we used LC–MS/MS to compare global protein expression in untreated MRSA_US14_ and phage-treated SCV MRSA_US14_ after 24 h of LB culture. At this time point, bacteriophage exposure induced a broad remodeling of the *S. aureus* proteome, with 177 proteins upregulated and 299 proteins downregulated compared to untreated controls. The magnitude of upregulation ranged from 1.16-fold to 6.72-fold (median: 1.384), whereas downregulation ranged from 1.16-fold to 19.09-fold (median: 1.573). The density plot (Figure 4A) illustrates a skew toward stronger downregulation, consistent with the extensive suppression of metabolic and stress-related pathways. PCA group separation (Figure 4B) was assessed using permutation-based PERMANOVA on Euclidean distances calculated from the scaled data (R^2^ = 0.40, *p* = 0.0078, 9999 permutations). No significant difference in multivariate dispersion was observed between groups (*p* = 0.7146), indicating that PERMANOVA results reflect centroid separation rather than differences in within-group variability. Overall, PCA revealed a clear segregation between control and phage-treated samples, confirming a global proteomic shift. Functional enrichment analysis highlighted distinct pathway-level responses. Upregulated proteins were significantly associated with ribosome biogenesis, aminoacyl-tRNA biosynthesis, and peptidoglycan biosynthesis, suggesting activation of translational machinery and cell-wall remodeling under phage stress (Figure 4C). In contrast, downregulated proteins mapped predominantly to central carbon metabolism, including glycolysis/gluconeogenesis, citrate cycle (TCA cycle), and glutathione metabolism (Figure 4D), indicating a metabolic downshift that likely reduces proton motive force. Literature-based analysis revealed several *S. aureus* proteins altered by phage treatment associated with β-lactam and aminoglycoside resistance (Figure 5A,B). Among the β-lactam resistance-related proteins, seven showed upregulation (5.15–1.26-fold), while six were downregulated (0.77–0.38-fold). For aminoglycoside resistance-related proteins, four were upregulated (1.49–1.32-fold) and 15 were downregulated (0.78–0.21-fold). These changes indicate substantial remodeling of resistance-associated protein expression.

### 2.5. Bacteriophage Exposure Decreases the Cell Wall Thickness of MRSA_US14_

**Figure 6:**
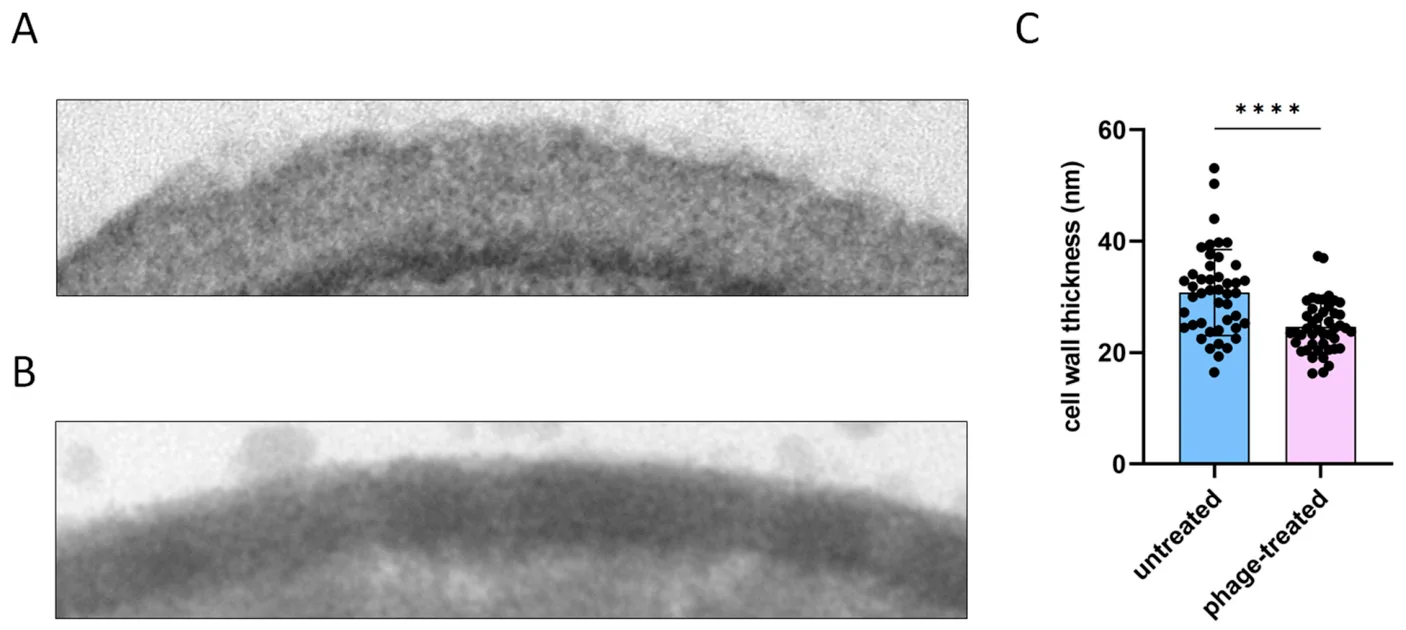
Ultrastructural analysis of the MRSA_US14_ cell wall after phage treatment. Representative transmission electron microscopy (TEM) images of MRSA_US14_ cells showing the cell wall in (**A**) untreated and (**B**) phage-treated cells. (**C**) Quantification of cell wall thickness of 15 representative untreated and phage-treated bacteria (three measurements in each bacterium) demonstrated a significant reduction in the phage-treated group compared with the untreated group. Individual dots represent independent measurements. Unpaired *t*-test **** *p* < 0.0001.

The genomic and proteomic alterations indicated substantial changes in pathways involved in cell-wall synthesis/ homeostasis following bacteriophage exposure; therefore, transmission electron microscopy (TEM) was performed to determine whether these molecular changes were accompanied by detectable ultrastructural alterations of the bacterial cell wall. To assess the ultrastructure of the bacterial envelope, the cell walls of untreated MRSA_US14_ and SCV MRSA_US14_ cells were examined by transmission electron microscopy. Representative images revealed an altered cell wall morphology in phage-treated bacteria compared with untreated controls (Figure 6A,B). Quantitative analysis confirmed that phage treatment significantly reduced cell wall thickness relative to the untreated group (Figure 6C). These findings indicate that phage treatment induces marked structural changes in the bacterial cell wall, consistent with cell envelope damage.

## 3. Discussion

In this study, we investigated the effect of a commercially available bacteriophage cocktail PYOFAG on a clinical MRSA isolate. The phage genome identified in the infected MRSA_US14_ assembly was a *Rosenblumvirus* SCH1. We showed that the bacteriophage treatment induced profound molecular and phenotypic changes, including the appearance of an SCV. *S. aureus* SCVs are slow-growing subpopulations with downregulated metabolism, which promote persistence and altered antimicrobial susceptibility [19]. SCV emergence can be triggered by stresses such as oxidative stress, acidic pH, cationic antimicrobial peptides, nutrient limitation, and antibiotic and bacteriophage exposure [20]. While SCVs are often linked to a persistent phenotype and muted antibiotic sensitivity, the most important phenotypic change in our study was a general increase in antibiotic sensitivity. Notably, besides SCV, larger colonies recovered after phage exposure were additionally observed to exhibit reduced antibiotic resistance, although their susceptibility profile differed from that of the SCV-like survivor population.

Comparison of the sequencing coverage depths of the bacteriophage and host genomes revealed that only a small fraction (~1.3%) of the host cells were infected. Furthermore, the average 45.6% variant allele frequency of several SNPs in the phage-exposed population indicates that the recovered survivors were genetically heterogeneous rather than descendants of a single resistant clone. This heterogeneity may reflect the selection of pre-existing resistant subpopulations, the parallel emergence of adaptive variants during phage exposure, or a combination of both. The fact that the observed absence of the two genomic regions was 15% of the assembly’s average coverage in the parental genome supports the idea that the parental strain was heterogeneous. Altogether, the observed genomic and proteomic differences predominantly characterize the heterogeneous surviving population rather than bacteria undergoing productive phage infection. A major phage resistance mechanism in *S. aureus* is mutations in genes involved in phage infection [21]. Among the mutations we identified, the most directly responsible for phage resistance are the ones disrupting TarS activity. TarS is a protein involved in wall teichoic acid (WTA) glycosylation, and its loss-of-function mutations change the WTA structure, preventing phage absorption. Therefore, TarS mutations are likely major drivers of phage resistance. A trade-off of phage resistance is increased susceptibility to antibiotics. This has been widely documented in the prokaryotic world. The WTA of *S. aureus* has a pivotal role in resistance against β-lactam antibiotics [22]; therefore, any loss-of-function mutation in WTA synthesis or modifying genes can reduce antibiotic resistance. The other mutations identified are auxiliary mutations involved in phage resistance or hitchhikers under phage selection pressure. The deleterious mutation in the PrmC protein slows down translation, which is beneficial under phage pressure; however, it causes a global slowdown of protein synthesis [23] and may have an indirect negative influence on antibiotic resistance. Amino acid changes disrupting the protein structure of surface protein SasG may also indirectly increase antibiotic susceptibility by slowing or restricting biofilm formation [24]. The above-mentioned mutations also negatively influence overall fitness and the virulence of the strain. The sequencing data showed that two genomic regions with a cumulative size of 18.2 kbp have been lost as a result of the selection pressure posed by the phage infection. This selection-driven genomic remodeling provides passive defense against viral infection in the surviving population. This phenomenon has been well documented as large-scale (up to several hundred kbp) genomic region loss in Gram-negative bacteria such as *Pseudomonas* and *Salmonella* [25,26,27]. In Gram-positive bacteria, genomic loss is less common and limited to several hundred base pairs of gene truncation [28]. To our knowledge, this is the first report of an ~18 kbp genomic loss in the *S. aureus* genome associated with adaptation under lytic bacteriophage selection pressure.

The larger genomic chunk lost encoded an entire operon of the type IV secretion system. The T4SS participates in bacterial conjugation and is a common target of phage infection via the conjugation pili in Gram-negative bacteria. However, the *Rosenblumvirus* SCH1 identified here uses cell wall teichoic acid as a receptor, not pili [29,30]. The coverage data in this region showed that the parental strain is also heterogeneous in terms of the T4SS presence. Thus, the absence of the T4SS operon likely reflects selection of a preexisting population rather than an active deletion event. We hypothesize that the deletion of the T4SS operon can indirectly contribute to phage resistance by affecting the cell surface structure. Although mutations that change the cell surface can impede phage infection in *S. aureus* [21], there is no direct experimental evidence so far that loss of the T4SS influenced the surface structure.

The phage-induced increased sensitivity was particularly notable against β-lactam antibiotics and aminoglycosides. While bacteriophage-induced genome alterations might not explain the increased antibiotic sensitivities alone, bacteriophage infection resulted in a significant change in the MRSA_US14_ proteome. The post-infection proteomic change included 466 significantly altered host proteins, representing ~17% of the *S. aureus* genome. The functional changes included various categories spanning pathways from energy metabolism to cell wall synthesis. Many of the altered proteins were linked previously to antibiotic resistance. Lytic phage infection perturbs cell-wall homeostasis by expressing endolysins to hydrolyze the peptidoglycan layer, leading to acute cell-wall damage and remodeling pressure [31]. Consistent with this, our proteomics data showed that the bacteriophage infection altered several proteins involved in cell-envelope biogenesis and remodeling, a determinant of β-lactam resistance in *Staphylococcus aureus* beyond mecA/PBP2a alone (interestingly, PBP2a was not among the significantly altered proteins). Phage infection downregulated multiple enzymes in peptidoglycan and teichoic acid synthesis (MurE, MurP, Asd, DapH, PanB/C, DltA, TarK), while simultaneously upregulated autolysins such as Atl, IsaA, and Sle1. This combination may weaken the structural integrity of the cell wall and increase autolytic activity. Because MRSA’s β-lactam resistance depends on proper wall teichoic acid scaffolding for PBP2a function, disruption of WTA pathways strongly sensitizes the bacterium to β-lactams [32]. In contrast, increases in MurA, Ddl, Pbp1, and Pbp4 expressions likely reflect compensatory reinforcement [33]. Importantly, downregulation of the envelope stress and homeostasis regulators VraR suggests diminished capacity to mount the canonical cell wall stress stimulon (VraSR) [34,35]. VraR is a response regulator that can induce a coordinated transcriptional program that enhances cell-wall repair, antibiotic tolerance, and survival under envelope-targeting stress. This stimulon represents a core adaptive response enabling *S. aureus* to rapidly counteract cell wall-active antibiotics. The increased aminoglycoside sensitivity could be a net phenotypic effect of pro- and anti-susceptibility pathways impacted by phage infection. Aminoglycoside uptake and lethality depend strongly on membrane potential (Δψ) [36]. Thus, the broad downregulation we observe in central metabolism such as glycolysis (e.g., down-regulated GapB, GpmA, GpmI, Fda, Pgk) may lead to limited aminoglycoside uptake and therefore increase resistance. On the other hand, it has been proposed that multiple classes of bactericidal antibiotics, including β-lactams, aminoglycosides, and fluoroquinolones, can promote reactive oxygen species (ROS)-associated damage, which may contribute to killing under some conditions [37]. In accordance with this, the phage-induced reduction in redox stress-related proteins, KatA, AhpC, AhpF, Bcp, TrxB, SodM, MsrA1 and MsrA2, suggests impaired peroxide detoxification and repair of oxidatively damaged proteins. Changes in transporter expression further reinforce multi-class sensitization. We observed decreased AbcA, an ABC transporter described as a β-lactam-associated persistence/survival factor in *S. aureus*; reduction in AbcA expression is consistent with increased intracellular exposure and reduced survival under antibiotic pressure [38]. Phage infection also altered the expression of numerous fatty-acid metabolism enzymes (AcpP↓, AcsA↓, MbcS↓, FabI/F/D↑), potentially leading to changes in membrane fluidity, charge, and lipid composition. Altered fatty-acid metabolism may affect membrane properties and thereby influence susceptibility to membrane-active agents and aminoglycoside uptake. The reduction in cell wall thickness observed after phage treatment provides ultrastructural support for the antibiotic susceptibility shift detected in our phenotypic assays. In MRSA, maintenance of a properly organized and sufficiently robust cell wall is essential for high-level β-lactam resistance; therefore, the thinning of the wall is consistent with the increased susceptibility to several β-lactam agents observed after phage exposure. These structural changes also agree with our genomic data, particularly the phage-selected alterations affecting *tarS*, a gene involved in wall teichoic acid glycosylation, which is known to be functionally linked to both phage adsorption and β-lactam resistance. In parallel, the proteomic results demonstrated broad remodeling of cell-envelope-associated pathways, including reduced abundance of several proteins involved in peptidoglycan and teichoic acid metabolism together with altered expression of autolysis-related factors. Taken together, these genomic, proteomic, and ultrastructural findings support a model in which phage-driven adaptation compromises cell wall homeostasis, thereby contributing to the re-sensitization of MRSA to selected antibiotics. Interestingly, despite the major biological differences between Gram-negative ESBL-producing *E. coli* in our previous study and Gram-positive MRSA in this study, bacteriophage exposure was associated with reduced antibiotic resistance in both organisms. One possible common contributing factor is the broad suppression of central metabolism and energy-generating pathways observed in both studies, which may reflect a shared fitness cost of adaptation to bacteriophage pressure.

## 4. Materials and Methods

### 4.1. Bacterium Isolate and Reagents

A clinical MRSA_US14_ isolate was obtained from routine clinical samples and stored in an anonymized strain collection of the Department of Medical Microbiology, University of Szeged. No patient-identifiable information was used. Cefoxitin disk diffusion testing on Mueller–Hinton agar, interpreted according to EUCAST 2025 criteria, was used as a phenotypic surrogate for the methicillin resistance phenotype, confirmed by identification of the *mecA* gene in the MRSA_US14_ genome using whole-genome sequencing. Species identification was carried out using a MALDI Biotyper system (Bruker Daltonics, Bremen, Germany). The PYOFAG bacteriophage cocktail (Neoprobio Care, Kyiv, Ukraine), containing more than 10^5^ phage particles per milliliter and active against *Escherichia coli*, *Streptococcus pyogenes*, *Staphylococcus aureus*, *Pseudomonas aeruginosa*, *Proteus vulgaris*, and *Proteus mirabilis*, was used in the experiments. Unless otherwise noted, all additional reagents were obtained from Sigma-Aldrich (St. Louis, MO, USA). Bacterial species were identified by matrix-assisted laser desorption/ionization time-of-flight mass spectrometry using the MALDI Biotyper platform (Bruker Daltonics, Bremen, Germany). The experiments were performed with the commercially available PYOFAG phage preparation (Neoprobio Care, Kyiv, Ukraine), which has a phage titer exceeding 10^5^ particles/mL and targets *Escherichia coli*, *Streptococcus pyogenes*, *Staphylococcus aureus*, *Pseudomonas aeruginosa*, *Proteus vulgaris*, and *Proteus mirabilis*. All other chemicals and reagents were purchased from Sigma-Aldrich (St. Louis, MO, USA), unless a different supplier is specified.

### 4.2. Antibiotic Susceptibility Tests and Growth Kinetics

MRSA_US14_ cultures were grown overnight in Luria–Bertani (LB) broth at 37 °C. The following day, the optical density at 600 nm (OD_600_) was measured and adjusted to 0.05. An aliquot of 80 µL of this standardized bacterial suspension (approximately 3.2 × 10^6^ CFU) was added to each well of a 96-well microtiter plate, followed by the addition of either 20 µL physiological salt or 20 µL of the bacteriophage cocktail. The plate was then incubated overnight at 37 °C in 5% CO_2_. After incubation, bacterial suspensions were streaked onto Columbia sheep blood agar plates (BioRad, Hercules, CA, USA) and incubated again overnight under identical conditions. The next day, isolated colonies of phage-treated SCV MRSA_US14_ and untreated MRSA_US14_ were used for antibiotic susceptibility testing. Susceptibility to amikacin (AKN), amoxicillin-clavulanate (AUG), ceftaroline (CPN), cefepime (FEP), ceftriaxone (CRO), cefoxitin (FOX), cefuroxime (CXM), ciprofloxacin (CIP), clindamycin (CLI), erythromycin (ERY), fusidic acid (FAD), gentamicin (GMN), linezolid (LIN), meropenem (MEM), moxifloxacin (MXF), mupirocin (PUM), norfloxacin (NXN), piperacillin-tazobactam (PTZ), quinupristin-dalfopristin (QDF), rifampicin (RIF), tetracycline (TET), tigecycline (TGC), trimethoprim-sulfamethoxazole (SXT), and tobramycin (TMN) was determined using the disk diffusion method. For growth kinetics, approximately 3.2 × 10^6^ CFU were incubated with the PYOFAG cocktail (20 µL) or left untreated for 1, 2, 3, 4, 5, 6, 7, and 24 h at 37 °C with 5% CO_2_ in LB broth. Optical density (OD_600_) was measured using an EZ Read 400 spectrophotometer (Biochrom, Cambridge, UK).

### 4.3. Genome Sequencing of Bacteriophage-Treated and Untreated MRSA_US14_

For genome sequencing, isolated colonies of untreated and small colony variant (SCV) MRSA_US14_ were used. Genomic DNA was extracted using the QIAamp DNA Mini Kit (Qiagen, Hilden, Germany) according to the manufacturer’s instructions. DNA concentrations were determined with the Qubit dsDNA HS Assay Kit on a Qubit 3.0 Fluorometer (Thermo Fisher Scientific, Waltham, MA, USA). Shotgun DNA-seq libraries were prepared with the NEXTFLEX Rapid XP DNA-Seq Kit v2 including unique dual indexes (PerkinElmer, Waltham, MA, USA) following the supplier’s protocol. Library concentrations were quantified using the Quant-it 1× dsDNA HS Assay Kit (Thermo Fisher Scientific, Waltham, MA, USA) and measured on a Fluostar Omega plate reader (BMG Labtech, Ortenberg, Germany). Library fragment size distributions were analyzed by capillary electrophoresis on a LabChip GX Touch HT Nucleic Acid Analyzer (PerkinElmer) using the X-Mark HT chip (CLS144006) and the DNA NGS 3k Assay Kit (CLS960013, PerkinElmer). The pooled libraries were diluted to 140 pM and sequenced using 2 × 150 bp paired-end reads with the 300-cycle 10B Reagent Kit on the NovaSeq X Plus System (Illumina, San Diego, CA, USA) according to the manufacturer’s instructions. Genomic DNA was isolated with the QIAamp DNA Mini Kit (Qiagen, Hilden, Germany) in accordance with the manufacturer’s protocol. DNA yield was assessed using the Qubit dsDNA HS Assay Kit and a Qubit 3.0 Fluorometer (Thermo Fisher Scientific, Waltham, MA, USA). Shotgun sequencing libraries carrying unique dual indexes were subsequently generated with the NEXTFLEX Rapid XP DNA-Seq Kit v2 (PerkinElmer, Waltham, MA, USA), following the supplier’s recommendations. Library concentrations were measured with the Quant-iT 1× dsDNA HS Assay Kit (Thermo Fisher Scientific, Waltham, MA, USA) on a FLUOstar Omega microplate reader (BMG Labtech, Ortenberg, Germany). Fragment-size profiles were evaluated by capillary electrophoresis using a LabChip GX Touch HT Nucleic Acid Analyzer equipped with an X-Mark HT chip (CLS144006) and the DNA NGS 3k Assay Kit (CLS960013; PerkinElmer, Waltham, MA, USA). After pooling, the libraries were adjusted to a final concentration of 140 pM and sequenced on a NovaSeq X Plus instrument (Illumina, San Diego, CA, USA) with the 300-cycle 10B Reagent Kit, generating 2 × 150 bp paired-end reads according to the manufacturer’s guidelines. Each sample yielded more than 5 Gbp of raw data. Demultiplexing was performed with NovaSeq Control Software v1.8, and adapter trimming as well as 3′/5′ base quality filtering (Q < 30) were completed using Trimmomatic 0.39 [39] with the following parameters: ILLUMINACLIP:2:30:10 SLIDINGWINDOW:4:20 MINLEN:100. Data quality was assessed using FastQC v0.12.1 [40]. The genomes of the untreated and phage-treated SCV strains were assembled separately with MEGAHIT v1.2.9 [41]. During assembly, mercy k-mers were eliminated using the no-mercy option, as recommended for generic datasets. Assembly statistics and quality were examined with QUAST v5.0.2 [42]. The assembly quality was confirmed with CheckM2 v1.0.1 [43]. Genomes were compared with Mauve 20150226 [44]. Genes were called with the help of Prokka v1.14.6 [45], and functional annotation was done using eggnog-mapper v2.1.12 [46] with eggNOG database version v5.0.2 [47], and running Diamond v2.0.15 [48] as an alignment method. Protein identifications were also confirmed using Prokka’s amino acid sequence output with BLAST v2.15.0 search [49] against the NCBI nr database, the CD-Search web application of the NCBI Conserved Domain Database [50] and the EMBL SMART protein identification web application [51]. For variant calling, the reads of the SCV MRSA_US14_ genome were first mapped to the untreated MRSA_US14_ assembly using BWA v0.7.19-r1273 with the BWA-MEM algorithm [52]. samtools v1.22.1 [53] was then used for mapping file conversion. Variants were called with FreeBayes v1.3.10 [54]. The VCF file was further processed using bcftools v1.22 [55], and the effect of mutations was predicted by snpEff v5.3a [56]. The phage genome was identified using the nucleotide BLAST algorithm on the NCBI webpage, searching in the core nucleotide database. The phylogenetic tree of the phage was created with the VICTOR web application [57]. The genome was also compared with the reference using Quast v5.0.2 and visualised with Circos v0.69-6 [58]. A list of phage-induced mutations in the MRSA_US14_ genome can be found in Supplementary Table S1. Raw sequencing reads can be found under the NCBI BioProject PRJNA1476909.

### 4.4. Proteome Analysis of Bacteriophage-Treated and Untreated MRSA_US14_ by Liquid Chromatography with Tandem Mass Spectrometry (LC–MS/MS)

For proteomic investigations, isolated colonies of untreated and SCV MRSA_US14_ (*n* = 5) were used. Bacterial cells were lysed using trifluoroacetic acid (TFA). 50 µL of 100% TFA was added to each pellet, incubated for 5 min at 55 °C, and subsequently neutralized with 450 µL of 2 M Tris (pH unadjusted). Protein concentration was determined by the BCA Protein Assay, following the manufacturer’s acetone precipitation procedure at a four-fold dilution. For protein digestion, 20 µg of total protein was subjected to reduction and alkylation (9 mM tris(2-carboxyethyl) phosphine and 40 mM chloroacetamide in TFA) for 5 min at 95 °C. Samples were then diluted with water to reach a final concentration of 1 M Tris and 5% TFA. Trypsin was added at a 1:50 enzyme-to-protein ratio, and digestion was carried out overnight at 30 °C. The reaction was terminated by adjusting the mixture to 3% formic acid, after which peptides were analyzed via LC–MS/MS using a Waters ACQUITY UPLC M-Class system coupled to an Orbitrap Exploris 240 mass spectrometer (Thermo Fisher Scientific, Waltham, MA, USA). Peptides were separated on a C18 analytical column under gradient elution conditions. Data were collected in a data-independent acquisition (DIA) mode, and raw files were processed with DIA-NN to construct a spectral library and quantify peptide abundances. Precursor identifications were filtered at a 1% false discovery rate (FDR), followed by differential expression analysis with FDR-corrected *p*-value thresholds. LC–MS data normalization and statistical analysis were performed in R using the MS-DAP package [59], applying the ‘vsn’ method for normalization and the ‘deqms’ algorithm for differential expression with an FDR < 0.05 significance cutoff. Only proteins detected in at least three samples with two unique peptides were included in the analysis. Functional annotation of significantly altered proteins was performed using DAVID software [60]. LC-MS/MS protein expression measurement of bacteriophage-treated SCV and control MRSA_US14_ can be found in Supplementary Table S2. For proteomic profiling, five biological replicates (*n* = 5) of isolated colonies from untreated MRSAUS_14_ and bacteriophage-exposed SCV MRSAUS14 cultures were analyzed. Cell pellets were lysed by adding 50 µL of neat trifluoroacetic acid (TFA), followed by incubation at 55 °C for 5 min. The lysates were subsequently neutralized with 450 µL of 2 M Tris without prior pH adjustment. Protein concentrations were measured using the BCA Protein Assay after acetone precipitation, as recommended by the manufacturer, with samples diluted fourfold. For enzymatic digestion, 20 µg of total protein from each sample was reduced and alkylated with 9 mM tris(2-carboxyethyl)phosphine and 40 mM chloroacetamide in TFA at 95 °C for 5 min. Water was then added to obtain final concentrations of 1 M Tris and 5% TFA. Proteolysis was performed overnight at 30 °C with trypsin at an enzyme-to-protein ratio of 1:50. Digestion was stopped by bringing the formic acid concentration to 3%. The resulting peptides were examined by LC–MS/MS using a Waters ACQUITY UPLC M-Class system connected to an Orbitrap Exploris 240 mass spectrometer (Thermo Fisher Scientific, Waltham, MA, USA). Peptide separation was achieved on a C18 analytical column using gradient elution. Mass-spectrometric data were acquired in data-independent acquisition (DIA) mode, and the resulting raw files were processed with DIA-NN for spectral-library generation and peptide quantification. Precursor-level identifications were restricted to a false discovery rate (FDR) of 1%, and differential protein abundance was evaluated using FDR-adjusted *p*-values. Normalization and statistical evaluation of the LC–MS dataset were conducted in R with the MS-DAP package [59]. Variance-stabilizing normalization (vsn) was applied, while differential abundance was assessed using the deqms algorithm with an FDR threshold of <0.05. Proteins were retained only when identified by at least two unique peptides in a minimum of three samples. Significantly altered proteins were functionally annotated using DAVID [60]. Protein-abundance data for the bacteriophage-exposed SCV and untreated MRSAUS14 groups are provided in Supplementary Table S2.

### 4.5. Transmission Electron Microscopy (TEM)

For TEM investigations, isolated colonies of untreated and SCV MRSA_US14_ were used. All specimens were received in 3% glutaraldehyde solution at the Department of Pathology. Samples were post-fixed in fresh 3% glutaraldehyde solution. After rinsing in phosphate-buffered saline (PBS), samples were further fixed for 1 h in 2% OsO_4_. After dehydrating in increasing ethanol concentrations, they were rinsed in uranyl acetate and acetone. Then the samples were embedded in Embed812 (Electron Microscopy Sciences, Hatfield, PA, USA). The embedded blocks were used to prepare ultrathin (70 nm) sections using an Ultracut S ultra-microtome (Leica, Wetzlar, Germany), which were then mounted on copper grids. The grids were counterstained with uranyl acetate and lead citrate and were examined and photographed with a JEOL JEM 1400 transmission electron microscope (JEOL; Tokyo, Japan). Digital photographs of bacterial cells were acquired and measured at a magnification of 150,000× with the TEM Center software and Sight XViewer (JEOL; Tokyo, Japan). Following rinsing with phosphate-buffered saline (PBS), the samples were post-fixed in 2% osmium tetroxide (OsO_4_) for 1 h. They were subsequently dehydrated through a graded ethanol series and treated with uranyl acetate and acetone before being embedded in Embed 812 resin (Electron Microscopy Sciences, Hatfield, PA, USA). Ultrathin sections approximately 70 nm in thickness were cut from the resin blocks with an Ultracut S ultramicrotome (Leica, Wetzlar, Germany) and collected on copper grids. The sections were contrast-stained with uranyl acetate and lead citrate and subsequently visualized using a JEOL JEM-1400 transmission electron microscope (JEOL, Tokyo, Japan). Digital micrographs were captured at 150,000× magnification, and bacterial cell measurements were performed using TEM Center and SightX Viewer software (JEOL, Tokyo, Japan).

## 5. Conclusions

In our study, bacteriophage exposure induced broad phenotypic and molecular remodeling in MRSA_US14_, including genomic, proteomic, and structural changes, with significant thinning of the cell wall and increased susceptibility to multiple antibiotics. These findings support the concept that phage pressure can generate evolutionary trade-offs that weaken clinically relevant resistance mechanisms. Given that this phenotype is likely driven by several interacting pathways, functional validation using targeted knockouts, complementation, and other mechanistic experiments will require separate, dedicated studies. Altogether, our results highlight the potential of bacteriophages not only as direct antibacterial agents but also as modulators of antibiotic susceptibility in multidrug-resistant *S. aureus*.

## Data Availability

Data supporting the reported results can be found in the Supplementary Materials. Raw sequencing reads can be found under the NCBI BioProject PRJNA1476909.

## Supplementary Materials

The following supporting information can be downloaded at: https://www.mdpi.com/article/10.3390/antibiotics15080800/s1, Table S1: List of phage-induced mutations in the MRSA_US14_ genome; Table S2: LC-MS/MS protein expression measurement of bacteriophage-treated SCV and control MRSA_US14_.
